# CMTM3 suppresses chordoma progress through EGFR/STAT3 regulated EMT and TP53 signaling pathway

**DOI:** 10.1186/s12935-021-02159-5

**Published:** 2021-09-24

**Authors:** Wanqiong Yuan, Feng Wei, Hanqiang Ouyang, Xiaoqing Ren, Jing Hang, Xiaoning Mo, Zhongjun Liu

**Affiliations:** 1 Department of Orthopedics, Peking University Third Hospital, 49 North Garden Road, Haidian District, Beijing, 100191 China; 2 Beijing Key Laboratory of Spinal Disease, Beijing, China; 3 Engineering Research Center of Bone and Joint Precision Medicine, Ministry of Education, Beijing, China; 4 Department of Pharmacy, Peking University Third Hospital, 49 North Garden Road, Haidian District, Beijing, China; 5 Center for Reproductive Medicine, Department of Obstetrics and Gynecology, Beijing, China; 6 Peking University Third Hospital, Key Laboratory of Assisted Reproduction, Ministry of Education, 49 North Garden Road, Haidian District, Beijing, 100191 China; 7 Beijing Key Laboratory of Reproductive Endocrinology and Assisted Reproduction, Beijing, China; 8 Department of Immunology, Key Laboratory of Medical Immunology, Ministry of Health, School of Basic Medical Sciences, Peking University Center for Human Disease Genomics, Peking University Health Science Center, 38 Xueyuan Road, Haidian District, Beijing, 100191 China

**Keywords:** Chordoma, CMTM3, EGFR, STAT3, TP53

## Abstract

**Background:**

Chordomas are rare, slow-growing and locally aggressive bone sarcomas. At present, chordomas are difficult to manage due to their high recurrence rate, metastasis tendency and poor prognosis. The underlying mechanisms of chordoma tumorigenesis and progression urgently need to be explored to find the effective therapeutic targets. Our previous data demonstrates that EGFR plays important roles in chordoma development and CKLF-like MARVEL transmembrane domain containing (CMTM)3 suppresses gastric cancer metastasis by inhibiting the EGFR/STAT3/EMT signaling pathway. However, the roles and mechanism of CMTM3 in chordomas remain unknown.

**Methods:**

Primary chordoma tissues and the paired adjacent non-tumor tissues were collected to examine the expression of CMTM3 by western blot. The expression of CMTM3 in chordoma cell lines was tested by Real-time PCR and western blot. CCK-8 and colony forming unit assay were performed to delineate the roles of CMTM3 in cell proliferation. Wound healing and Transwell assays were performed to assess cell migration and invasion abilities. A xenograft model in NSG mice was used to elucidate the function of CMTM3 in vivo. Signaling pathways were analyzed by western blot and IHC. RNA-seq was performed to further explore the mechanism regulated by CMTM3 in chordoma cells.

**Results:**

CMTM3 expression was downregulated in chordoma tissues compared with paired normal tissues. CMTM3 suppressed proliferation, migration and invasion of chordoma cells in vitro and inhibited tumor growth in vivo. CMTM3 accelerated EGFR degradation, suppressed EGFR/STAT3/EMT signaling pathway, upregulated TP53 expression and enriched the TP53 signaling pathway in chordoma cells.

**Conclusions:**

CMTM3 inhibited tumorigenesis and development of chordomas through activating the TP53 signaling pathway and suppressing the EGFR/STAT3 signaling pathway, which suppressed EMT progression. CMTM3 might be a potential therapeutic target for chordomas.

**Supplementary Information:**

The online version contains supplementary material available at 10.1186/s12935-021-02159-5.

## Background

Chordomas, derived from embryonic remnants of the notochord, are rare and slow-growing primary malignant tumors of the spine with an incidence rate of 1–4% of all bone cancers [1, 2]. The major treatment for chordomas is surgery and postoperative radiotherapy as chordomas are refractory to cytotoxic chemotherapeutics [3]. However, it is difficult to treat chordomas due to their local destructive behavior and their preference for growing near the critical skull base, lumbosacral nerves and blood vessels [4]. In addition, high recurrence (> 50% of patients) [5] and metastasis are major causes of treatment failure in chordomas [6, 7]. Herein, it is necessary to study the mechanism of chordoma tumorigenesis and progression and find the effective therapeutic targets for chordoma treatment.

Epidermal growth factor receptor (EGFR), a member of tyrosine kinase receptors, is generally overexpressed in chordomas [8]. EGFR affects tumor growth and metastasis [9]. Our previous study shows that phosphorylated EGFR plays important roles in chordomas progress [10]. EGFR phosphorylation triggers downstream signaling pathways such as signal transducer and activator of transcription 3 (STAT3) [11], AKT [12] and ERK1/2 [13] pathways. The activation of these signaling pathways further drives epithelial–mesenchymal transition (EMT) [14], which is a critical process in cancer metastasis [15, 16]. However, only a few studies demonstrate that EMT is involved in the pathogenesis of chordomas [17]. STAT3 activation also decreases the expression of TP53 and suppresses the TP53 signaling pathway [18], which also responds to stress signals and regulates the expression of its target genes leading to various cellular responses to prevent tumorigenesis [19, 20] as a transcription factor. At present, only Ma’ group explicitly proposes that the TP53 (alias: p53) signaling pathway plays a role in chordoma development [21]. It is necessary and crucial to clarify how the EGFR and TP53 signaling pathway make contributions to chordoma tumorigenesis and progression.

CKLF-like MARVEL transmembrane domain containing (CMTM)3 is a member of the CMTM family [22, 23]. It is located on 16q22.1, an important tumor suppressor locus with tumor suppressive properties [24]. CMTM3 is frequently reduced in multiple types of cancer, such as prostate cancer [25, 26] and renal cell carcinoma [27]. Moreover, CMTM3 exhibits tumor-suppressor roles in cancer progression, including testicular cancer [28], oral squamous cell carcinoma [29] and gastric cancer [30]. Our previous study demonstrates that CMTM3 suppresses metastasis of gastric cancer through the STAT3/EMT signaling pathway by interacting with Rab5 to facilitate EGFR degradation [31, 32] and CMTM3 may serve as a prognostic and predictive biomarker in gastric cancer [33]. However, whether CMTM3 participates in the tumorigenesis and development of chordomas remain unknown.

In this paper, we investigate the effects and mechanism of CMTM3 in chordomas. First, we find that CMTM3 is downregulated in chordoma tissues compared with the paired normal tissues. Second, functional experiments reveal that CMTM3 suppresses chordoma cells proliferation, migration and invasion in vitro and inhibits the chordoma tumor growth in vivo. Third, a mechanistic study shows that CMTM3 facilitates EGFR degradation, suppresses EGFR/STAT3 signaling pathway-regulated EMT progression and activates TP53 signaling pathway in chordoma cells. This study will help us understand the pathogenesis of chordomas and provide us with a potential target for the treatment of chordomas.

## Methods

### Patients and tissue specimens

The study cohort consisted of eight chordoma patients including four males and four females, who had no preoperative treatment and who had undergone uneventful surgical resection (mean age: 55.25-years old, range, 31–71-years old) between 2019 and 2020 in Peking University Third Hospital. None of the patients received neoadjuvant chemotherapy or radiation therapy and all patients underwent surgery at our department. The work described has been carried out in accordance with The Code of Ethics of the World Medical Association (Declaration of Helsinki). Informed consent was obtained from each patient. The site of each tumor is listed in Table 1. Paired distant normal muscle tissues were collected at least 5 cm from the tumor margins as controls. Fresh human tissues were frozen in liquid nitrogen for protein extraction.

**Table 1:** Tumor site of the eight chordomas patients

Patient number	Tumor site
Tumor 1	Sacral vertebrae
Tumor 2	Cervical vertebra 2
Tumor 3	Cervical vertebra 2–6
Tumor 4	Thoracic vertebrae 2
Tumor 5	Cervical vertebra 2–5
Tumor 6	Cervical vertebra 2
Tumor 7	Thoracic vertebrae 9- lumbar vertebra 2
Tumor 8	Cervical vertebra 6

### Cell lines and reagents

Human chordoma U-CH1, MUG-Chor1 and JHC7 cell lines were purchased from American Type Culture Collection (ATCC, Manassas, VA, USA). U-CH1 and MUG-Chor1 cells were cultured in RPMI-1640 medium (Catalog No. 11875093, Gibco, US) supplemented with an additional 1% l-glutamine (Catalog No. 25030081, Gibco, US), 10% characterized fetal bovine serum (FBS) (Catalog No. 10099141, Gibco, US), 10 units/mL penicillin and 10 mg/mL streptomycin (Catalog No. 10378016, Gibco, US). JHC7 cells were cultured in DMEM: F12 medium (ATCC^®^ 30-2006™, ATCC, USA) supplemented with 10% FBS, 10 units/mL penicillin and 10 mg/mL streptomycin. To culture U-CH1 and MUG-Chor1 cells, coating buffer (50 μg/mL rat tail type I collagen (Catalog No. 354236, BD Biosciences) was added to the culture flask for 1 h at room temperature prior to adding the cells. The SGC-7901 (a gastric cancer cell line)-shN and sh393 cells and GES-1 (an immortalized gastric epithelial cell line)-shN and sh393 cells were obtained as previously described [32]. All cells were maintained in humidified incubators at 37 °C with 5% CO_2_.

### Reverse transcription (RT) and Real-time PCR

RT-PCR was performed as previously described [31]. Briefly, total RNA was extracted from cells or tissues using TRIzol reagent (Catalog No. 15596018, Invitrogen, USA) and cDNA was generated using a First-Strand cDNA Synthesis Kit (Catalog No. K1622, Invitrogen, USA) according to standard protocols. For Real-time PCR, amplifications were performed using a SYBR Green PCR Master Mix Kit (Catalog No. KK4610, Roche, USA) according to the manufacturer’s instructions. The mRNA expression was calculated using the 2−ΔΔCt method. β-actin expression was used as a control. All reactions were repeated in triplicate. Primers were shown in Table 2.

**Table 2:** Primer sequences for qPCR

Name	Primer sequences
CMTM3	F: 5ʹ-ACCGCGGCCCTCATCTACT-3ʹ
	R: 5ʹ-AGGCCTTCAGTCAGAGTCC-3ʹ
EGFR	F: 5ʹ-TCTCAGCAACATGTCGATGG-3ʹ
	R: 5ʹ-TCGCACTTCTTACACTTGCG-3ʹ
TP53	F: 5ʹ-GCAAAACATCTTGTTGAGGGC-3ʹ
	R: 5ʹ-CCAGGGCAGCTACGGTTTC-3ʹ
HSPA6	F: 5ʹ-GATGTGTCGGTTCTCTCCATTG-3ʹ
	R: 5ʹ-CTTCCATGAAGTGGTTCACGA-3ʹ
β-actin	F: 5ʹ-CCAACCGCGAGAAGATGA-3ʹ
	R: 5ʹ-CCAGAGGCGTACAGGGATAG-3ʹ

*F* Forward primer, *R* Reverse primer

### Western blot and antibodies

Western blot was performed as previously described [31]. Antibodies against EGFR (Catalog No. 4267), phosphor-EGFR (Tyr1068) (Catalog No. 3777), ERK1/2 (Catalog No. 4695), phosphor-ERK1/2 (Thr202/Tyr204) (Catalog No. 4376), AKT (pan) (Catalog No. 4691), phosphor-AKT (Ser473) (Catalog No. 4060), STAT3 (Catalog No. 9139), phosphor-STAT3 (Tyr705) (Catalog No. 9145), N-cadherin (Catalog No. 13116), Vimentin (Catalog No. 5741), TP53 (Catalog No. 2527) were purchased from CST company (MA, USA) and E-cadherin (Catalog No. 610404) were purchased from BD company. The dilution of the above antibodies was 1:1000. The rabbit anti-CMTM3 antibody was prepared and purified in our laboratory [24] and was used at a dilution of 1:800 (working concentration: 2 μg/mL). To detect the endogenous CMTM3 expression, 100 μg of total protein lysates were loaded, and 40 μg of total protein lysates were loaded for the detection of other proteins.

### Immunohistochemistry (IHC)

Immunohistochemistry was performed on formalin-fixed, paraffin-embedded tissues from mice as previously described [32].

### Adenovirus construction and cell infection

The construction, generation, purification and infection of the ad5-null (vector-containing empty adenovirus, defined as MOCK) and ad5-CMTM3 vectors were produced by Hanbio Biotechnology Co., Ltd. (Shanghai, China) and MOCK was used in parallel as a negative control. Cells were infected with MOCK or Ad-CMTM3 at a multiplicity of infection (MOI) value of 100. After 2 days, the infected cells were collected for subsequent experiments.

### siRNA transfection

To knock down endogenous CMTM3, small interfering RNA (siRNA) constructs were generated with the following target sequences: si-CMTM3-1^#^, 5ʹ-GCAACUGAUUUCUACCUGATT-3ʹ, si-CMTM3-2^#^, 5ʹ-UUAACGACGUGGCCAAAUUTT3ʹ. Scrambled siRNA (Scr) was used as a negative control with the sequence: 5ʹ-UUCUCCGAACGUGUCACGUTT-3ʹ. The siRNAs were purchased commercially (Gene Pharma Inc., Shanghai, China). Chordoma cells were transfected with siRNA at a final concentration of 50 nM using Lipofectamine 3000 transfection reagent (Catalog No. L3000015, Invitrogen, USA). The suppression efficiency of CMTM3 was analyzed by western blot 3 days post transfection.

### Counting Kit-8 (CCK-8) assay

Cell proliferation was measured by a CCK-8 detection kit (Catalog No. CK04, Dojindo Molecular Technologies, Japan). The cells were seeded in a 96-well plate with 3000 cells per well in the CMTM3-overexpressed systems and 2000 cells per well in the CMTM3-silenced systems [34, 35]. At the indicated time points, 10 μL of CCK-8 solution was added to each well followed by incubation at 37 °C for 2 h, and absorbance at 450 nm was determined.

### Colony forming unit (CFU) assay

The cells were seeded into a 6-well plate at a density of 1000 cells per well for CMTM3 overexpressed JHC7 cells and U-CH1 cells, 500 cells per well for CMTM3 silenced U-CH1 cells [36] and 1000 cells per well for CMTM3 silenced MUG-Chor1 cells [34]. Complete culture medium was replaced twice a week. After 2 weeks, the cell colonies were fixed in 4% paraformaldehyde/PBS and stained with 2% crystal violet. The number of colonies (≥ 50 cells per colony) was counted in five randomly chosen fields. Each assay was performed in triplicate.

### Wound-healing assay

Cells were seeded in a 6-well plate (at a density of 2 × 10^5^ cells/mL [37]) and a wound was scraped with a sterilized pipette tip in the middle of the cell monolayer and grown to 80% confluence. Photomicrographs were taken on day 0 and day 3 in the CMTM3 overexpressed systems and on day 0 and day 2 in the CMTM3 silenced systems. ImageJ software (National Institutes of Health, USA) was used to measure wound width at different time points. The percentage of wound healing was calculated from the initial wound width at 0 h.

### Cell migration and invasion assay

Cell migration and invasion assays were performed with 8-μm Transwell chambers (Catalog No. 3422, Corning, USA). The cells were cultured in serum-free medium overnight before the initiation of the experiments. For migration assay, 1 × 10^5^ cells in 0.25 mL of serum-free medium were seeded into the upper chamber [38] and 0.5 mL of medium with 10% FBS was added to the lower chamber and incubated at 37 °C for 24 h. For the invasion assay, 70 μL of 1:10-chilled serum-free medium-diluted Matrigel (Catalog No. 354234, BD Biosciences, NJ, USA) was precoated in the upper chamber and incubated in a humidified incubator at 37 °C for 60 min. Next, 2 × 10^5^ cells [39] in 0.25 mL of serum-free medium were seeded into the upper chamber with Matrigel and 0.5 of mL medium with 10% FBS was added to the lower chamber. After incubated at 37 °C for 48 h, the chambers were disassembled, and the membranes were fixed in 4% paraformaldehyde for 10 min and stained with 2% crystal violet for 10 min. The number of cells was counted in five randomly chosen fields and images were obtained using a microscope (100× magnification).

### Establishment of the chordoma mouse model

To establish the subcutaneous chordoma mouse model, 5- to 6-week-old female NOD-SCID interleukin 2 receptor gamma (NSG) null mice (Vital River, Beijing, China) were used. The mice were bred and maintained under specific pathogen free conditions, provided with sterilized food and water and housed in a barrier facility with a 12 h light/dark cycle. The mice were randomly divided into two groups (n = 6) and were inoculated with 6 × 10^6^ U-CH1-MOCK cells or U-CH1-CMTM3 cells, respectively. The cells were centrifuged and resuspended in PBS mixed with Matrigel at a 1:1 ratio to a final volume of 200 μL and subcutaneously inoculated into the flanks of mice. After 5 months, all mice were sacrificed by cervical dislocation and visible tumors were photographed and weighed. Tumors were measured along two orthogonal axes (a = length, b = width) and tumor volume was calculated using the following formula: volume = a × b^2^/2 [40].

### EGFR degradation assay

Cells were serum starved for 16 h and then treated with 25 µg/mL Cycloheximide (CHX) (Catalog No. 40325ES03, Yeasen, Shanghai, China) for 2 h and 100 ng/mL EGF (Catalog No. AF-100-15, Pepro Tech, USA) for different time points. At the end of each time point, the cells were washed with PBS and then lysed in lysis buffer. The lysates were subjected to SDS-PAGE and immunoblotting with appropriate antibodies.

### Confocal microscopy

For the detection of the colocalization between CMTM3 and endogenous EGFR, pEGFP-N1 empty vector or pEGFP-N1-CMTM3 plasmid was transfected into JHC7 cells. After 48 h, the cells were washed, fixed, permeabilized, incubated with primary antibodies overnight at 4 °C and incubated with Alexa594-conjugated secondary antibodies. Next, the cells were washed three times with PBS and stained with DAPI (Catalog No. B1061, Applygen, China) for 10 min before imaging with a TCS-SP laser scanning confocal microscope (Leica Microsystems, Mannheim, Germany).

### Co-immunoprecipitation (Co-IP) assay

Co-immunoprecipitation assay was performed using a protocol as previously described [32]. Briefly, cells were lysed with the cell lysis buffer. 1 mg protein were incubated with 4 µg of the anti-EGFR antibodies (Catalog No. 4267, CST, USA) overnight at 4 ℃ under rotation. Then, they were incubated with 30 µL protein G Sepharose (Catalog No. 17061801, GE, USA) at 4 ℃ for 2 h under rotation. The beads were washed three times with the lysis buffer and resuspended in SDS sample buffer, boiled for 10 min, and analyzed by western blot.

### RNA sequencing (RNA-seq) and data analysis

Adenovirus was used to overexpress CMTM3 in JHC7 cells that were seeded at a density of 3 × 10^5^ cells per well in six-well plates. TRIzol reagent was used to extract total RNA by a standard protocol. DNA contamination was removed by Turbo DNase treatment (Life Technologies) and RNA was purified using a RNeasy Mini Kit (Catalog No. 74903, Qiagen, USA). After extraction, RNA purity was checked using the NanoPhotometer^®^ spectrophotometer (IMPLEN, CA, USA). RNA integrity was measured by the RNA 6000 Bioanalyzer Nano Kit of the Bioanalyzer 2100 system (Agilent Technologies, CA, USA) according to the manufacturer’s instructions. RNA samples with a minimum RIN score of 7.8 were used for further analyses.

A total 3 µg of RNA per sample was used as input material for the RNA sample preparations. Sequencing libraries were generated using the NEBNext^®^ Ultra™ RNA Library Prep Kit for Illumina^®^ (Catalog No. E7770, NEB, USA) following the manufacturer’s recommendations and index codes were added to attribute sequences to each sample. Briefly, mRNA was purified from total RNA using poly-T oligo-attached magnetic beads. Fragmentation was carried out using divalent cations under elevated temperature in NEBNext First Strand Synthesis Reaction Buffer (5×). First strand cDNA was synthesized using random hexamer primers and M-MuLV Reverse Transcriptase (RNase H-). Second strand cDNA synthesis was subsequently performed using DNA Polymerase I (Catalog No. 18010017, Invitrogen, USA) and RNase H (Catalog No. AM2292, Invitrogen, USA). The remaining overhangs were converted into blunt ends by exonuclease/polymerase activities. After adenylation of 3ʹ ends of DNA fragments, NEBNext adaptors with hairpin loop structures were ligated to prepare for hybridization. The library fragments were purified with the AMPure XP system (Beckman Coulter, Beverly, USA) to preferentially select cDNA fragments of 250–300 bp. 3 µL of USER Enzyme (Catalog No. M5505S, NEB, USA) was used with size-selected, adaptor-ligated cDNA at 37 °C for 15 min followed by 5 min at 95 °C before PCR. PCR was performed with Phusion High-Fidelity DNA polymerase, universal PCR primers and Index (X) Primer. Finally, PCR products were purified (AMPure XP system) and library quality was assessed with the Agilent Bioanalyzer 2100 (Agilent Technologies, Santa Clara, CA, USA).

Feature Counts v1.5.0-p3 was used to count the read numbers mapped to each gene. FPKM, which is the number of fragments per kilobase of transcript sequence per millions base pairs sequenced, considers the effect of sequencing depth and gene length on the read count at the same time, and is currently the most commonly used method for estimating gene expression levels. The FPKM of each gene was calculated based on the length of the gene and the read count mapped to the gene.

Differential expression analysis between JHC7-MOCK and JHC7-CMTM3 was performed using the DESeq2 R package (1.16.1). The resulting p-values were adjusted using Benjamini and Hochberg’s approach for controlling the false discovery rate. Genes with an adjusted p-value (padj) < 0.05 found by DESeq2 were considered to be as differentially expressed.

KEGG is a database resource for understanding the high-level functions and utilities of biological systems, such as cells, organisms and ecosystems, from molecular-level information, especially large-scale molecular datasets generated by genome sequencing and other high-throughput experimental technologies (http://www.genome.jp/kegg/). The Cluster Profiler R package was used to test the statistical enrichment of differentially expressed genes in KEGG pathways. Differences were considered significant at padj < 0.05.

### Statistical analysis

The results of three independent experiments are presented as the mean ± s.d. Statistical analysis was carried out with Student’s t-test in Prism 6.0 (GraphPad Software, San Diego, CA, USA) between two groups. p-values < 0.05 (two-sided) were considered statistically significant (ns, not significant, *p < 0.05, **p < 0.01, ***p < 0.001). Gray values that were obtained by western blot were analyzed by ImageJ software.

## Results

### CMTM3 expression is reduced in chordoma tissues and suppresses the proliferation of chordoma cells

**Fig. 1:**
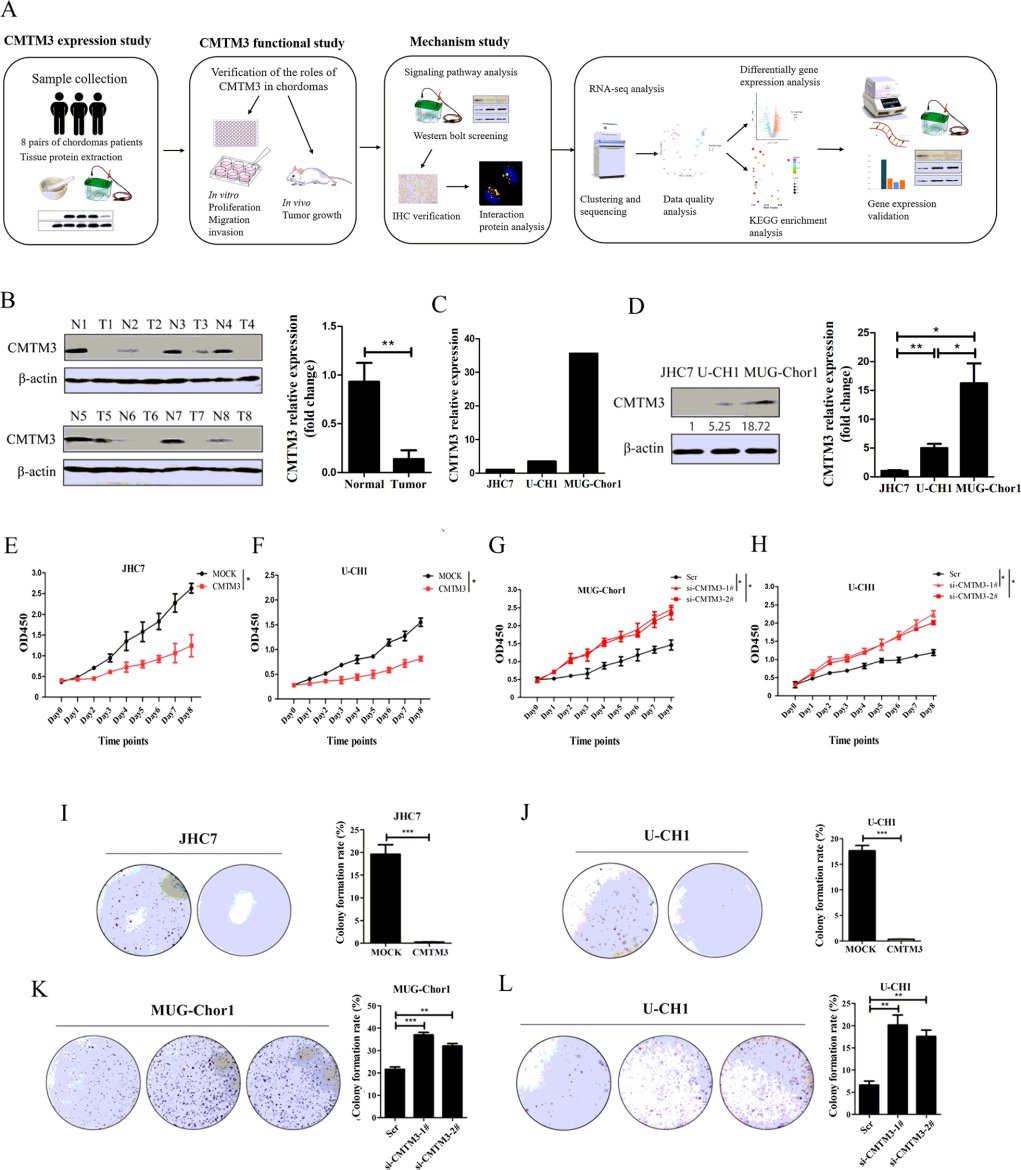
CMTM3 was downregulated in chordoma tissues and inhibited the growth of chordoma cells. **A** The general research methodology of the entire study. **B** The expression of CMTM3 in tumor (T) and paired adjacent non-tumor (N) tissues was detected by western blot. **C** The expression of CMTM3 in chordoma cell lines was detected by Real-time PCR. Representative data was shown as the average number from one independent experiment. **D** The expression of CMTM3 in chordoma cell lines was detected by western blot. **E**–**H** CCK-8 assays were performed in chordoma cells after CMTM3 overexpression or silencing. **I**–**L** CFU assays of chordoma cells in CMTM3 overexpressed or silenced cells (**I**–**K**: 1000 cells/well. **L**: 500 cells/well). Data are representative of three independent experiments and presented as the mean ± s.d (Scr: control siRNA, *p < 0.05, **p < 0.01, ***p < 0.001)

The procedure of the entire study was illustrated in Fig. 1A, which included three parts: the CMTM3 expression study, the functional study and the mechanism study. Our previous study showed that CMTM3 was downregulated in gastric cancer patients and gastric cancer cells, and acted as a tumor suppressor in gastric cancer development [31, 32]. In this study, the expression of CMTM3 was analyzed in eight paired chordoma tissues and their adjacent normal tissues by western blot. As shown in Fig. 1B, CMTM3 expression was significantly downregulated in the tumor tissues compared with the matched adjacent normal tissues at the protein level, which suggested that CMTM3 might play important roles in chordoma tumorigenesis and development. Then, the expression of CMTM3 in chordoma JHC7, U-CH1, MUG-Chor1 cell lines was analyzed by Real-time PCR. The CMTM3 expression level was lowest in JHC7 cells, followed by U-CH1 and MUG-Chor1 (Fig. 1C). We further confirmed the expression level of chordoma cell lines by western blot and obtained consistent results (Fig. 1D).

To identify whether CMTM3 participates in tumorigenesis, we analyzed the proliferation of chordoma cells. We used adenovirus to overexpress CMTM3 in chordoma cell lines expressing relatively low levels of endogenous CMTM3 (JHC7 and U-CH1). To study the endogenous role of CMTM3, we knocked down CMTM3 expression by siRNA in U-CH1 and MUG-Chor1 cells. CCK-8 assays revealed that overexpression of CMTM3 inhibited cell growth in JHC7 and U-CH1 cells (Fig. 1E, F). Silencing CMTM3 promoted cell proliferation in MUG-Chor1 and U-CH1 cells (Fig. 1G, H). Likewise, CFU assays further confirmed that CMTM3 suppressed chordoma proliferation (Fig. 1I–L). These data revealed that CMTM3 suppressed the proliferation of chordoma cells.

### CMTM3 inhibits the migration and invasion of chordoma cells

**Fig. 2:**
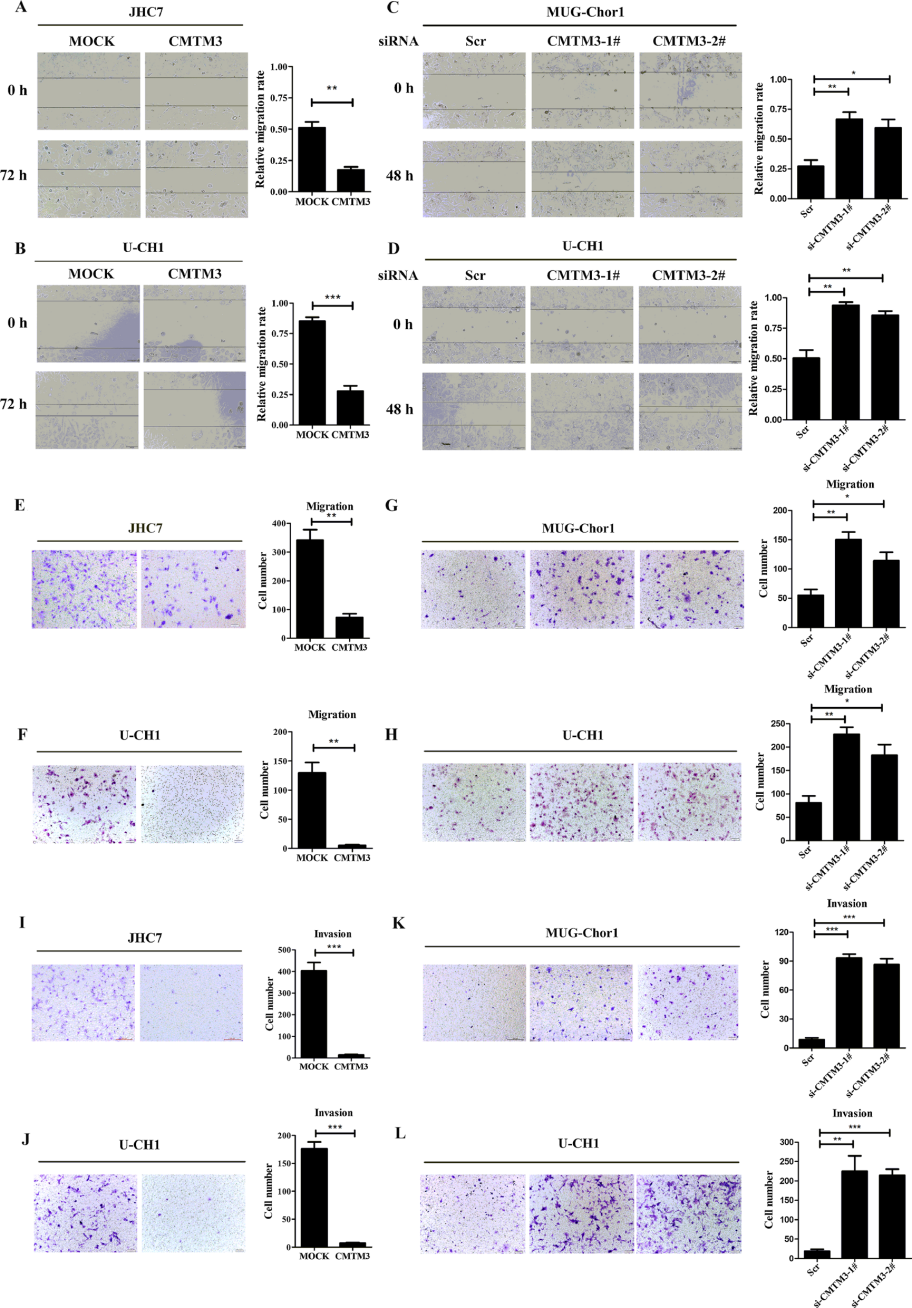
CMTM3 inhibited migration and invasion of chordoma cells. **A**–**D** Wound healing assays of chordoma cells in CMTM3 overexpressed (72 h) or silenced chordoma cells (48 h) (100× magnification). **E**–**H** Transwell assays of cell migration in CMTM3 overexpressed or silenced chordoma cells. **I**–**L** Transwell assays of cell invasion in CMTM3 overexpressed or silenced chordoma cells (100× magnification). Data were representative of three independent experiments and presented as the mean ± s.d (Scr: control siRNA, *p < 0.05, **p < 0.01, ***p < 0.001)

To explore the role of CMTM3 in chordoma development, cell migration and invasion were detected after modifying the expression of CMTM3. Wound-healing assays were performed in CMTM3-overexpressed JHC7 (Fig. 2A) and U-CH1 cells (Fig. 2B) and CMTM3-silenced MUG-Chor1 (Fig. 2C) and U-CH1 cells (Fig. 2D). We observed that the wound edge of the MOCK cells was markedly closer than that of the CMTM3 overexpressed cells and the wound edge of the CMTM3 silenced cells was closer than that of the Scr cells. Similarly, Transwell assays showed that CMTM3 suppressed cell migration in chordoma cells (Fig. 2E–H). Transwell assays were then performed to evaluate the invasion of CMTM3 in chordoma cells precoated with Matrigel in the chamber and we found that overexpressed CMTM3 remarkably inhibited invasion of JHC7 cells (Fig. 2I) and U-CH1 cells (Fig. 2J), and knockdown of CMTM3 revealed the opposite effects in MUG-Chor1 (Fig. 2K) and U-CH1 cells (Fig. 2L). Taken together, CMTM3 suppressed the migration and invasion of chordoma cells in vitro.

### CMTM3 suppresses tumor growth in vivo

**Fig. 3:**
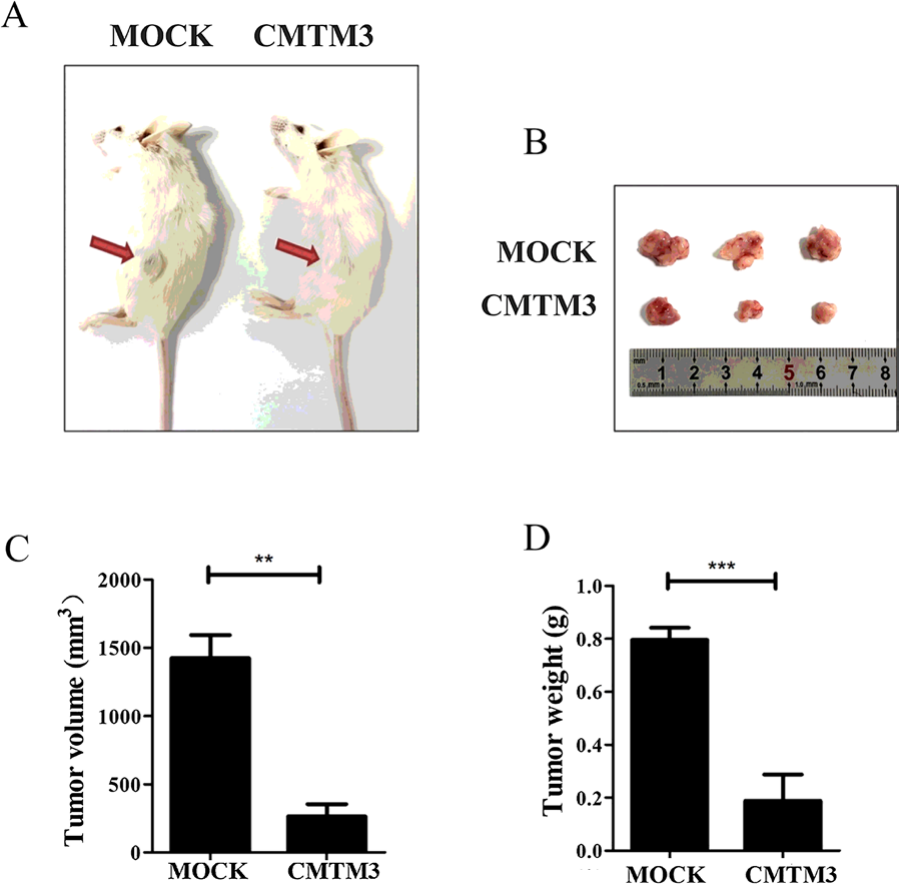
CMTM3 inhibited tumor growth in vivo. A total of 6 × 10^6^ cells (U-CH1-MOCK or U-CH1-CMTM3) were inoculated into 5- to 6-week-old female NSG mice (n = 6 per group). **A** Images of tumor-bearing mice and **B** Visible tumors were obtained after the mice were sacrificed 5 months later. **C** The tumor volume of the mice was measured, calculated and shown in the bar graphs. **D** Tumor weight was measured and shown in the bar graphs

We have demonstrated that CMTM3 inhibited the proliferation, migration and invasion of chordoma cells in vitro. We subsequently employed a mouse model of chordomas to analyze the effects of CMTM3 in vivo by subcutaneously inoculating the flanks of NSG mice with U-CH1-MOCK cells or U-CH1-CMTM3 cells. The mice were sacrificed by cervical dislocation 5 months after cell inoculation and the tumors were photographed (Fig. 3A, B). The tumor volume (Fig. 3C) and tumor weight (Fig. 3D) were decreased in the CMTM3 group mice in comparison to that in the MOCK group mice. These data suggested that CMTM3 inhibited tumor growth in vivo.

### CMTM3 facilitates EGFR degradation and suppresses the EGFR/STAT3/EMT signaling pathway in chordoma cells

**Fig. 4:**
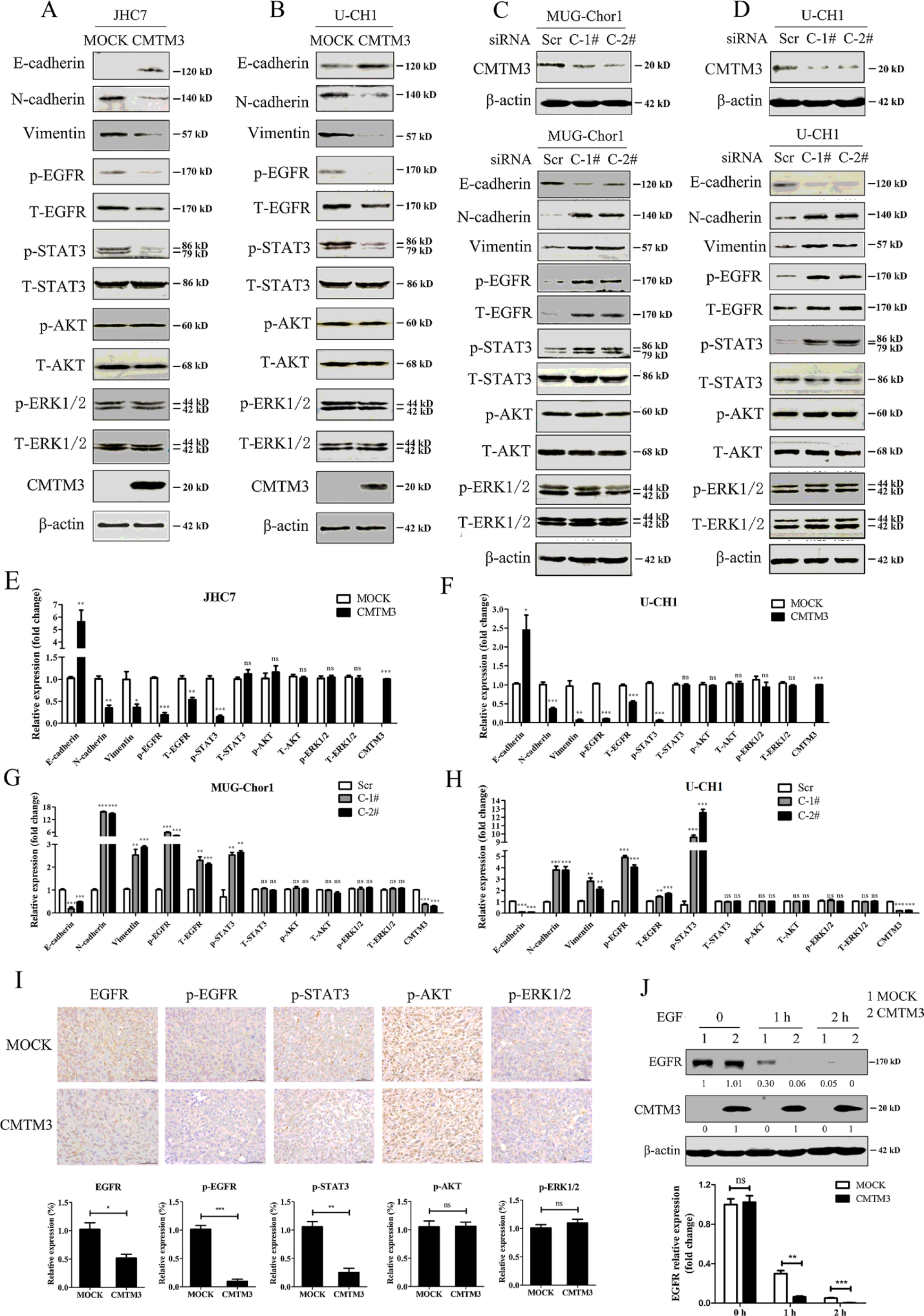
CMTM3 suppressed the EGFR/STAT3/EMT signaling pathway. **A**, **B** The expression of EMT-related markers and the phosphorylated and total EGFR, STAT3, AKT and ERK1/2 were analyzed in CMTM3 overexpressed chordoma cells by western blot. **C**, **D** Up, the transfection efficiency of the CMTM3 siRNAs was detected by western blot in MUG-Chor1 and U-CH1 cells, respectively (Scr: control siRNA, C-1^#^: CMTM3-1^#^, C-2^#^: CMTM3-2^#^). 100 μg of total protein lysates were loaded; Down, expression of EMT related markers: E-cadherin, N-cadherin and Vimentin, and the phosphorylated and total EGFR, STAT3, AKT and ERK1/2 were analyzed in CMTM3 silenced chordoma cells by western blot. Data were representative of three independent experiments. **E**, **F** The relative gray density of the indicated proteins shown in **A**, **B**, respectively (statistical analysis was carried out with CMTM3 vs. MOCK). **C**, **D** The relative gray density of the indicated proteins shown in **C**, **D**, respectively (statistical analysis was carried out with C-1# vs. Scr and C-2# vs. Scr). **I** Representative images are shown of IHC staining to assess the expression levels of EGFR, p-EGFR, p-STAT3, p-AKT and p-ERK1/2 in tumors from the mouse model of chordomas (*p < 0.05, **p < 0.01, ***p < 0.001). **J** Chordoma cells were treated with EGF (100 ng/mL) in the presence of CHX (25 μg /mL) for the indicated times and the cell lysates were subjected to immunoblotting with the indicated antibodies (MOI: 100)

EMT is a critical process in the metastatic cascade in many types of cancers [41]. Our previous studies found that CMTM3 participated in EMT in gastric cancer cells [31]. To illuminate the mechanism of the suppressive roles of CMTM3 in chordomas, we detected the expression of the epithelial specific junction protein E-cadherin and the mesenchymal proteins N-cadherin and Vimentin in CMTM3-overexpressed JHC7 and U-CH1 cells and CMTM3 silenced MUG-Chor1 and U-CH1 cells. As shown in Fig. 4A, B, overexpression of CMTM3 suppressed EMT along with upregulating E-cadherin expression and downregulating N-cadherin and Vimentin expression. Meanwhile, we knocked down CMTM3 in MUG-Chor1 (Fig. 4C, up) and U-CH1 cells (Fig. 4D, up) by siRNA and found that knockdown of CMTM3 induced EMT progress (Fig. 4C, D, down). EMT is triggered by different signaling cascades, such as the EGFR/STAT3, EGFR/AKT and EGFR/ERK1/2 signaling pathways. Therefore, we tested the levels of total and phosphorylated p-EGFR (Tyr1068), p-STAT3 (Tyr705), p-AKT (Ser473) and p-ERK1/2 (Thr202/Tyr204) in chordoma cells by western blot. As revealed in Fig. 4A, B, overexpression of CMTM3 decreased EGFR expression level, p-EGFR and p-STAT3, but no significant differences were observed in p-AKT or p-ERK1/2 in chordoma cells as analyzed in Fig. 4E, F. Moreover, knockdown of CMTM3 increased EGFR expression level, p-EGFR and p-STAT3 as shown in Fig. 4C, D, and analyzed in Fig. 4G, H. To further confirm the above results, we used IHC staining to analyze EGFR expression level. p-EGFR, p-STAT3, p-AKT and p-ERK1/2 in tumor samples from the chordoma mice model. The expression level of EGFR was reduced in U-CH1-CMTM3 mice, less p-EGFR and p-STAT3 were observed in U-CH1-CMTM3 mice, and no significant differences in p-AKT or p-ERK1/2 were observed between the two groups (Fig. 4I).

In addition, we detected EGFR expression in CMTM3 overexpressed JHC7 and U-CH1 cells and CMTM3 silenced MUG-Chor1 cells by Real-time PCR and found that CMTM3 did not alter EGFR expression at the mRNA level (Additional file 1A), indicating CMTM3 might facilitate EGFR degradation in chordoma cells. Next, we detected the EGFR degradation by CHX treatment and found that overexpression of CMTM3 facilitated EGFR degradation with EGF stimulation for 1 and 2 h (Fig. 4J). These data suggest that CMTM3 downregulates EGFR expression by promoting EGFR degradation.

To explore how CMTM3 influenced EGFR degradation and its downstream signaling pathway, we detected the interaction between EGFR and CMTM3 by confocal microscopy to observe the colocalization between GFP-CMTM3 and endogenous EGFR in JHC7 cells. We observed that CMTM3 colocalized with EGFR (Additional file 1B). However, CMTM3 was not immunoprecipitated with the anti-EGFR antibody by Co-IP assay (Additional file 1C). These results suggested that CMTM3 did not interact with EGFR. Taken together, CMTM3 suppresses chordomas metastasis via accelerating EGFR degradation and suppressing the EGFR/STAT3/EMT signaling pathway.

### CMTM3 suppressed the proliferation of chordoma cells by activating the TP53 signaling pathway

**Fig. 5:**
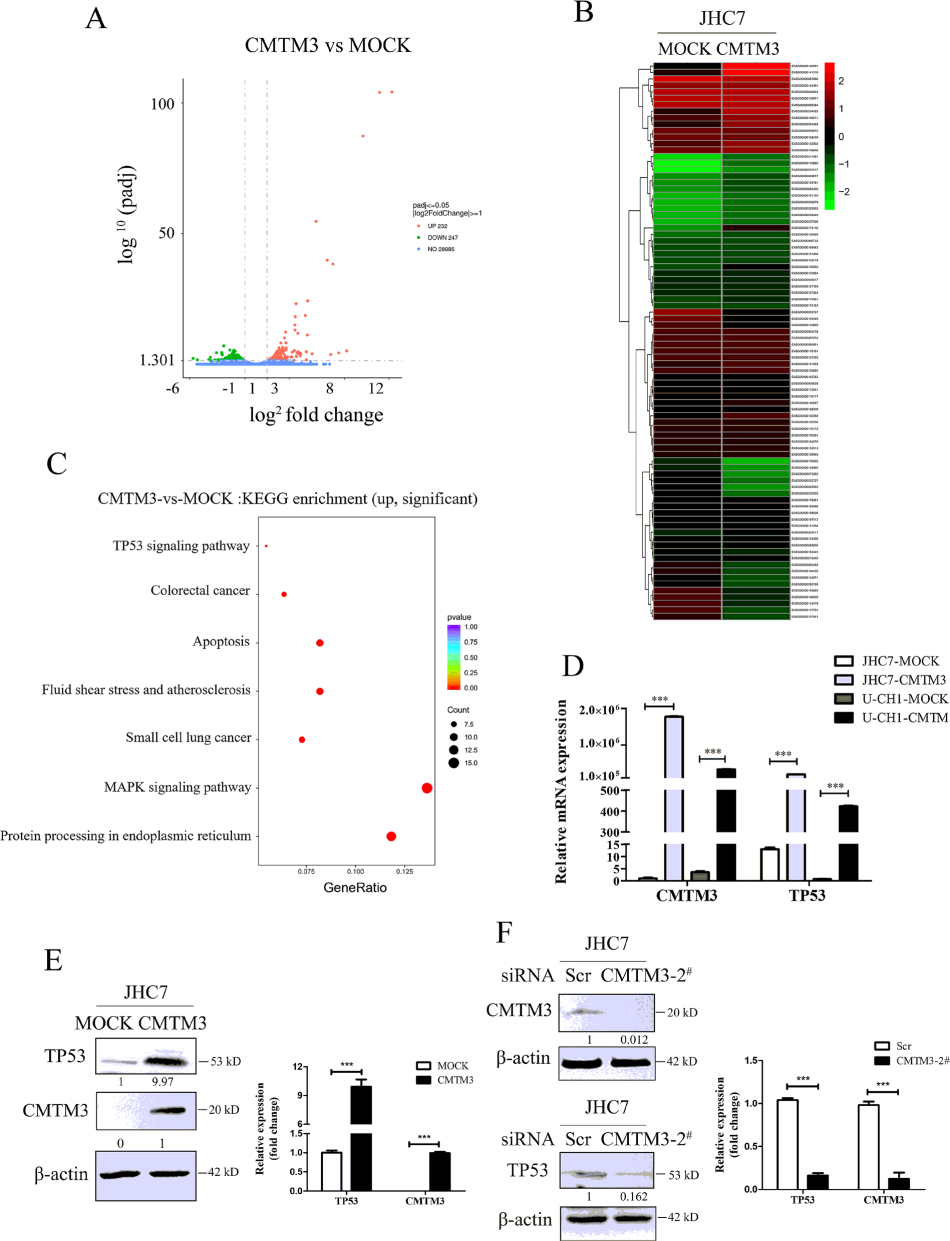
CMTM3 induces changes in gene expression profiles. **A** The number of different expressed genes (padj < 0.05, MOI: 100). **B** A heat map summary of the gene expression values of JHC7-MOCK and JHC7-CMTM3 cells. Red indicates high and green indicates low gene expression values (padj < 0.001). **C** KEGG enrichment analysis. The TOP seven upregulated signaling pathways are shown of CMTM3-vs-MOCK in JHC7 cells (padj < 0.05). **D** The expression of TP53 was detected in CMTM3 overexpressed chordoma cell lines by Real-time PCR. **E** The expression of TP53 was detected in CMTM3 overexpressed JHC7 cells by western blot. **F** Up, the transfection efficiency of the CMTM3-siRNA was detected in JHC7 cells by western blot. 100 μg of total protein lysates were loaded; Down. the expression of TP53 in CMTM3 silenced JHC7 cells was detected by western blot. Data were representative of three independent experiments

CMTM3 suppressed the migration and invasion of chordoma cells through the EGFR/STAT3/EMT signaling pathway. The pathways involved in the suppressed proliferation of chordoma cells driven by CMTM3 remain unknown. RNA-Seq technology is a transcriptomic sequencing technique and provides information about the count level of transcripts [42]. We overexpressed CMTM3 using adenovirus and investigated the whole transcriptome of JHC7 cells using Illumina RNA-Seq technology to screen the potential signaling pathway involved in CMTM3-suppressed cell proliferation. The results showed that there were 479 differently expressed genes (DEGs) between JHC7-MOCK and JHC7-CMTM3 cells with 232 upregulated genes and 247 downregulated genes (padj < 0.05, Fig. 5A, Additional files 2, 3). Among these DEGs, 68 genes were significantly upregulated and 18 genes were downregulated (Table 3, Fig. 5B, padj < 0.001).

**Table 3:** 68 upregulated and 18 downregulated genes in JHC7-CMTM3 cells vs. JHC7-MOCK cells (padj < 0.001)

Gene ID	Gene name	Gene description	Log^2^ fold change (CMTM3/MOCK)	padj
Upregulated DEGs				
ENSG00000140931	CMTM3	CKLF like MARVEL transmembrane domain containing 3	12.25	6.90E−105
ENSG00000141510	TP53	Tumor protein p53	11.14	9.02E−105
ENSG00000173110	HSPA6	Heat shock protein family A (Hsp70) member 6	9.65	4.80E−88
ENSG00000106211	HSPB1	Heat shock protein family B (small) member 1	5.42	2.11E−55
ENSG00000204388	HSPA1B	Heat shock protein family A (Hsp70) member 1B	6.41	1.53E−40
ENSG00000204389	HSPA1A	Heat shock protein family A (Hsp70) member 1A	6.93	5.06E−39
ENSG00000132002	DNAJB1	DnaJ heat shock protein family (Hsp40) member B1	4.66	6.02E−25
ENSG00000149257	SERPINH1	Serpin family H member 1	3.61	7.00E−24
ENSG00000105550	FGF21	Fibroblast growth factor 21	4.44	2.35E−19
ENSG00000151929	BAG3	BCL2 associated athanogene 3	3.53	6.39E−19
ENSG00000120694	HSPH1	Heat shock protein family H (Hsp110) member 1	3.50	9.56E−16
ENSG00000109846	CRYAB	Crystallin alpha B	3.51	8.51E−14
ENSG00000130513	GDF15	Growth differentiation factor 15	2.66	5.81E−13
ENSG00000080824	HSP90AA1	Heat shock protein 90 alpha family class A member 1	3.77	1.56E−12
ENSG00000100292	HMOX1	Heme oxygenase 1	4.62	7.00E−12
ENSG00000004478	FKBP4	FK506 binding protein 4	2.56	9.83E−11
ENSG00000109971	HSPA8	Heat shock protein family A (Hsp70) member 8	2.70	7.19E−09
ENSG00000130766	SESN2	Sestrin 2 [Source:HGNC Symbol;Acc:HGNC:20746]	2.24	8.27E−09
ENSG00000099875	MKNK2	MAP kinase interacting serine/threonine kinase 2	2.39	3.77E−07
ENSG00000100591	AHSA1	Activator of HSP90 ATPase activity 1	2.07	5.39E−07
ENSG00000188483	IER5L	Immediate early response 5 like	2.72	6.14E−07
ENSG00000168439	STIP1	Stress induced phosphoprotein 1	2.35	3.19E−06
ENSG00000168209	DDIT4	DNA damage inducible transcript 4	2.71	3.41E−06
ENSG00000174951	FUT1	Fucosyltransferase 1 (H blood group)	2.45	3.48E−06
ENSG00000115541	HSPE1	Heat shock protein family E (Hsp10) member 1	2.14	3.58E−06
ENSG00000128965	CHAC1	ChaC glutathione specific gamma-glutamylcyclotransferase 1	2.56	3.90E−06
ENSG00000116161	CACYBP	Calcyclin binding protein	2.16	4.86E−06
ENSG00000224411	HSP90AA2P	Heat shock protein 90 alpha family class A member 2, pseudogene	3.45	6.23E−06
ENSG00000178381	ZFAND2A	Zinc finger AN1-type containing 2A	1.98	8.53E−06
ENSG00000110680	CALCA	Calcitonin related polypeptide alpha	8.18	8.98E−06
ENSG00000087074	PPP1R15A	Protein phosphatase 1 regulatory subunit 15A	2.47	9.76E−06
ENSG00000175084	DES	Desmin	2.42	9.94E−06
ENSG00000086061	DNAJA1	DnaJ heat shock protein family (Hsp40) member A1	2.01	9.94E−06
ENSG00000121068	TBX2	T-box 2	2.47	1.06E−05
ENSG00000162783	IER5	Immediate early response 5	1.91	1.06E−05
ENSG00000121769	FABP3	Fatty acid binding protein 3	2.16	1.06E−05
ENSG00000096384	HSP90AB1	Heat shock protein 90 alpha family class B member 1	2.18	1.43E−05
ENSG00000161180	CCDC116	Coiled-coil domain containing 116	4.54	1.75E−05
ENSG00000258469	CHMP4BP1	Charged multivesicular body protein 4B pseudogene 1	3.80	2.01E−05
ENSG00000028277	POU2F2	POU class 2 homeobox 2	3.27	2.38E−05
ENSG00000110172	CHORDC1	Cysteine and histidine rich domain containing 1	2.21	3.93E−05
ENSG00000008517	IL32	Interleukin 32	2.40	4.39E−05
ENSG00000139269	INHBE	Inhibin subunit beta E	3.70	4.39E−05
ENSG00000087086	FTL	Ferritin light chain	2.01	4.95E−05
ENSG00000144381	HSPD1	Heat shock protein family D (Hsp60) member 1	1.98	5.26E−05
ENSG00000225217	HSPA7	Heat shock protein family A (Hsp70) member 7	7.46	5.27E−05
ENSG00000101255	TRIB3	Tribbles pseudokinase 3	2.20	5.27E−05
ENSG00000116717	GADD45A	Growth arrest and DNA damage inducible alpha	1.79	5.27E−05
ENSG00000149781	FERMT3	Fermitin family member 3	3.99	5.98E−05
ENSG00000183696	UPP1	Uridine phosphorylase 1	1.80	6.76E−05
ENSG00000064300	NGFR	Nerve growth factor receptor	3.84	1.04E−04
ENSG00000164045	CDC25A	Cell division cycle 25A	1.93	1.05E−04
ENSG00000200879	SNORD14E	Small nucleolar RNA, C/D box 14E	5.05	1.10E−04
ENSG00000148926	ADM	Adrenomedullin	2.24	1.20E−04
ENSG00000167513	CDT1	Chromatin licensing and DNA replication factor 1	2.02	1.20E−04
ENSG00000282855	AC093591.3	INTS3 and NABP interacting protein (INIP) pseudogene	4.03	1.43E−04
ENSG00000123358	NR4A1	Nuclear receptor subfamily 4 group A member 1	2.01	1.74E−04
ENSG00000211891	IGHE	Immunoglobulin heavy constant epsilon	6.74	1.96E−04
ENSG00000131094	C1QL1	Complement C1q like 1	1.69	2.43E−04
ENSG00000105219	CNTD2	Cyclin N-terminal domain containing 2	2.56	3.06E−04
ENSG00000088826	SMOX	Spermine oxidase	1.76	4.19E−04
ENSG00000068028	RASSF1	Ras association domain family member 1	1.89	4.19E−04
ENSG00000175183	CSRP2	Cysteine and glycine rich protein 2	2.22	5.01E−04
ENSG00000213430	HSPD1P1	Heat shock protein family D (Hsp60) member 1 pseudogene 1	2.20	5.33E−04
ENSG00000237596	AL138828.1	Novel transcript	3.98	6.21E−04
ENSG00000127564	PKMYT1	Protein kinase, membrane associated tyrosine/threonine 1	2.14	7.75E−04
ENSG00000066735	KIF26A	Kinesin family member 26A	2.13	9.19E−04
ENSG00000164070	HSPA4L	Heat shock protein family A (Hsp70) member 4 like	1.99	9.97E−04
Downregulated DEGs				
ENSG00000147724	FAM135B	Family with sequence similarity 135 member B	− 2.91	1.04E−07
ENSG00000147041	SYTL5	synaptotagmin like 5	− 2.36	3.81E−06
ENSG00000155850	SLC26A2	Solute carrier family 26 member 2	− 2.01	5.79E−06
ENSG00000103489	XYLT1	Xylosyltransferase 1	− 1.88	1.15E−05
ENSG00000203727	SAMD5	Sterile alpha motif domain containing 5	− 1.97	1.61E−05
ENSG00000146938	NLGN4X	Neuroligin 4 X-linked	− 2.06	2.08E−05
ENSG00000176055	MBLAC2	Metallo-beta-lactamase domain containing 2	− 3.08	3.93E−05
ENSG00000073282	TP63	Tumor protein p63	− 3.57	4.30E−05
ENSG00000232727	YWHAEP1	Tyrosine 3-monooxygenase/tryptophan 5-monooxygenase activation protein epsilon pseudogene 1	− 2.16	1.04E−04
ENSG00000114019	AMOTL2	Angiomotin like 2	− 2.01	1.20E−04
ENSG00000143971	ETAA1	ETAA1, ATR kinase activator	− 2.04	2.23E−04
ENSG00000082482	KCNK2	Potassium two pore domain channel subfamily K member 2	− 1.67	2.40E−04
ENSG00000225980	OR7E19P	Olfactory receptor family 7 subfamily E member 19 pseudogene	− 3.05	2.43E−04
ENSG00000280798	LINC00294	Long intergenic non-protein coding RNA 294	− 1.78	4.11E−04
ENSG00000138380	CARF	Calcium responsive transcription factor	− 2.40	5.01E−04
ENSG00000143669	LYST	lysosomal trafficking regulator	− 1.56	6.21E−04
ENSG00000250305	TRMT9B	tRNA methyltransferase 9B (putative)	− 2.14	7.52E−04
ENSG00000104432	IL7	Interleukin 7	− 1.62	8.32E−04

We observed that the expression of the well-known tumor suppressor gene TP53, a target of STAT3 was obviously unregulated by overexpression of CMTM3 (ranking first besides CMTM3). In addition, The KEGG pathway enrichment analysis of JHC7 cells showed that the upregulated TP53 was involved in most of the top significant enrichment signaling pathways (padj < 0.05), particularly the TP53 signaling pathway, MAPK signaling pathway and apoptosis in CMTM3 overexpressed JHC7 cells (Fig. 5C). Wherein, TP53 signaling pathway plays important roles in regulation of tumorigenesis and progression and the expression of genes involved in the TP53 signaling pathway was significantly upregulated in CMTM3 overexpressed JHC7 cells, including TP53, SESN2, GADD45A, TP73, GADD45B and CYCS (Table 3, Fig. 5B).

Then, we confirmed these results with JHC7, U-CH1 and MUG-Chor1 cells by Real-time PCR and revealed that TP53 expression was apparently upregulated in CMTM3 overexpressed cells (Fig. 5D). Western blot was further performed in JHC7 cells and the expression of TP53 was upregulated in CMTM3 overexpressed cells (Fig. 5E). We knocked down CMTM3 in JHC7 cells (Fig. 5F, up) and found that TP53 was downregulated in CMTM3 silenced cells (Fig. 5F, down) at the protein level. Our previous study demonstrated that CMTM3 suppressed migration and invasion, but not the proliferation of gastric cancer cells [31, 32]. To clarify whether TP53 upregulation modulated CMTM3 inhibited cell proliferation, we analyzed TP53 expression in gastric cancer cell line SGC-7901 and the gastric epithelial cell line GES-1 by CMTM3 knockdown system using lentivirus transduction [31]. As shown in Additional file 4, TP53 expression was not influenced by CMTM3 knocked down in SGC-7901 and GES-1 cells. These data suggested that CMTM3 might contribute to inhibiting cell proliferation by upregulating TP53 expression. It has been reported that TP53 may respond to stress signals [43] and we found that the expression of heat shock protein family A member 6 (HSPA6), a member of the heat shock protein family was (ranking second besides CMTM3) was upregulated (Fig. 5B, Table 3). Additional file 5 further confirmed that the expression of HSPA6 was upregulated by CMTM3 at the mRNA level by Real-time PCR. These results suggested that TP53 signaling pathway involved in the CMTM3-suppressed proliferation in chordoma cells.

Taken together, we propose that CMTM3 suppresses chordomas through EGFR/STAT3 mediated EMT progression and TP53 signaling pathway.

## Discussion

At present, the major treatment for chordomas is surgery and postoperative radiotherapy and they are difficult to be treated due to their destructive behavior locally and their sites [4]. Besides, it is crucial to accurately assess the dose profile outside the tumor with radiation treatment [44]. Therefore, it is necessary to explore the mechanism of chordomas tumorigenesis and development, and find its effective therapeutic targets. It has been reported that *CMTM3* acts as a tumor suppressor gene to inhibit numerous cancers. Our previous data also demonstrated that CMTM3 suppressed the metastasis of gastric cancer through the EGFR/STAT3/EMT signaling pathway by interacting with Rab5 [31, 32]. In this paper, we used CMTM3 overexpressed and CMTM3 silenced systems to explore the roles and mechanism of CMTM3 in chordomas. This is the first time we found that CMTM3 inhibited chordoma with regulating EGFR/STAT3 mediated EMT progression and the TP53 signaling pathway. This study investigates the function and mechanism of CMTM3 in chordomas and provides us with a potential target for the treatment of chordomas. In the future, we may characterize the tumor tissues and cells by Raman-enhanced spectroscopy probe [45] to identify CMTM3 for potential tumor markers or diagnosis.

EGFR plays critical roles in the progression of cancers. Strong EGFR expression is significantly associated with tumor growth and metastasis [8]. Adle-Biassette demonstrated that EGFR was expressed in 244/284 (85.9%) of chordomas [46] and Yilmaz found that intensely positive EGFR was revealed in 77.5% (38/49) of chordoma patients [47]. Our previous study also found that EGFR played important roles in chordoma tumorigenesis and development [10]. In this paper, we found that CMTM3 facilitated the degradation of EGFR, and reduced EGFR expression level and p-EGFR (Fig. 4). CMTM3 colocalized with EGFR, but CMTM3 was not coimmunoprecipitated with the anti-EGFR antibody by Co-IP assay (Additional file 1B, C), which suggested that CMTM3 did not interact with EGFR. To date, the clinical relevance of EGFR in chordoma development is still controversial because of its relatively low incidence rate. Inhibitors [48, 49] or antibodies [50] against EGFR may act as potential treatments for chordomas. However, clinical trials with EGFR inhibitors and antibodies have not established a clear therapeutic benefit in all chordoma patients [51, 52]. We believe it is necessary and interesting to explore whether CMTM3 participates in facilitating the sensitivity to EGFR-targeted drugs and antibodies.

As we mentioned, CMTM3 inhibited tumorigenesis and progression mostly in epithelial-derived cancers and few studies revealed the roles of CMTM3 in mesenchymal-derived cancers. To our knowledge, this is the first study to assess the effects and mechanism of CMTM3 in mesenchymal cell-derived tumors. Our previous studies revealed that CMTM3 inhibited cell migration, but did not affect cell proliferation in epithelial-derived gastric cancer. In addition, CMTM3 reduced the phosphorylation of ERK1/2 in gastric cancer cells [32]. In this paper, CMTM3 inhibited cell proliferation and migration, but did not influence ERK1/2 phosphorylation in mesenchymal-derived chordomas. Thus, the roles of CMTM3 might vary in different cell-derived tumors.

TP53 plays crucial roles in tumor initiation and progression [53, 54]. This is the first time we revealed that CMTM3 regulates the TP53 signaling pathway in tumorigenesis and tumor progression. In this study, CMTM3 inhibited cell proliferation and suppressed p-STAT3, which significantly upregulated TP53 expression and enriched the TP53 signaling pathway in chordoma cells. However, CMTM3 has no effects on gastric cancer cell proliferation and does not alter TP53 expression after silencing CMTM3 by lentivirus (Additional file 4). These results suggest that CMTM3 suppresses cancer cell proliferation through upregulation of TP53 expression and activation of the TP53 signaling pathway.

Although our findings demonstrate the roles and mechanism of CMTM3 in the tumorigenesis and development of chordomas, this study still has limitations. First, we revealed that CMTM3 suppressed chordoma cell proliferation, migration and invasion in vitro, and tumor growth in vivo. However, tumor metastasis was not observed in the tumor-bearing mice. We speculated that this is because of the slow-growing characteristics of chordomas and that 5 months were not enough for tumor metastasis or for the observation of the chordoma-bearing mice survival. Second, we observed that CMTM3 reduced EGFR protein level and suppressed p-EGFR, and the fold change of p-EGFR is higher than that of EGFR (Fig. 4). Nevertheless, we still could not conclude that CMTM3 activated p-EGFR directly because the inhibition of EGFR phosphorylation may be a reflection of the reduced EGFR protein level.

## Conclusions

In summary, our study first shows the function and mechanism of CMTM3 in chordomas. We find that CMTM3 expression is reduced in chordoma tissues and inhibits proliferation, migration and invasion of chordoma cells in vitro and suppresses tumor growth in vivo. Furthermore, CMTM3 facilitates EGFR degradation and inhibits tumorigenesis and development of chordomas via EGFR/STAT3 regulated EMT signaling pathway and TP53 signaling pathway. CMTM3 may act as a promising molecular target for chordomas, which would be clinically beneficial.

## Supplementary Information

**Additional file 1.** A. The expression of EGFR in CMTM3 overexpressed JHC7 cells and U-CH1 cells and CMTM3 knocked-down MUG-Chor1 cells was detected by Real-time PCR. Representative data were shown as average number from one independent experiment. B. JHC7 cells were transfected with pEGFP-N1 empty vector or pEGFP-N1-CMTM3 plasmid for 48 h prior to fixation in 4% PFA and immunostained with antibodies against EGFR. EGFR staining is shown in red. Nuclei were visualized by DAPI (blue). The yellow color indicates the colocalization. The Pearson’s and Manders’ overlap coefficients were derived with LEICA QWin software (bar, 25 μm). C. Co-IP assay was performed with anti-EGFR antibody in CMTM3 overexpressed JHC7 cells and followed by western blot with the antibodies indicated (MOI: 100).

**Additional file 2. **CMTM3 induces changes in gene expression profiles. A heat map summary reflecting gene expression values of JHC7-MOCK and JHC7-CMTM3 cells (MOI:100) (columns). Red indicates high and green indicates low gene expression values (padj < 0.05).

**Additional file 3. **Upregulated and downregulated genes in JHC7-CMTM3 in comparison to JHC7-MOCK cells (padj < 0.05).

**Additional file 4. **The expression of TP53 was detected by Real-time PCR in CMTM3 knocked-down SGC-7901 and GES-1 cells. Representative data were shown as average number from one independent experiment.

**Additional file 5. **Overexpression of CMTM3 increased HSPA6 expression. The expression of HSPA6 in CMTM3 overexpressed JHC7 cells was determined by Real-time PCR. Representative data were shown as average number from one independent experiment.

## Data Availability

The datasets used or analyzed during the current study are available from the corresponding author on reasonable request.

## Abbreviations

CCK8: Counting Kit-8
CFU: Colony forming unit
CMTM: CKLF-like MARVEL transmembrane domain containing
Co-IP: Co-immunoprecipitation
DEGs: Differently expressed genes
EGFR: Epidermal growth factor receptor
EMT: Epithelial–mesenchymal transition
HSPA6: Heat shock protein family A member 6
IHC: Immunohistochemistry
MOI: Multiplicity of infection
RT: Reverse transcription
Scr: Scrambled siRNA
siRNA: Small interfering RNA
STAT3: Signal transducer and activator of transcription 3
