# Supplementary figures and images for: CMTM3 suppresses chordoma progress through EGFR/STAT3 regulated EMT and TP53 signaling pathway
Source: Cancer Cell Int. 2021 Sep 24;21:510. doi: 10.1186/s12935-021-02159-5 (PMC8461898; doi:10.1186/s12935-021-02159-5)

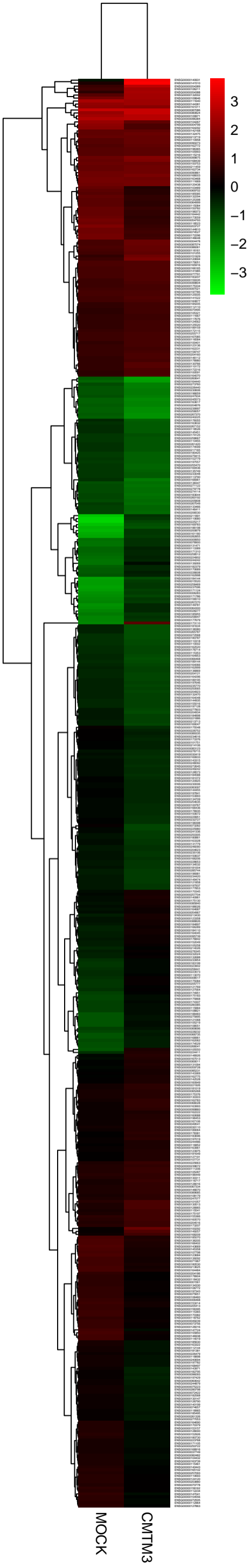

Supplement: Supplementary file 2 — Additional file 2. CMTM3 induces changes in gene expression profiles. A heat map summary reflecting gene expression values of JHC7-MOCK and JHC7-CMTM3 cells (MOI:100) (columns). Red indicates high and green indicates low gene expression values (padj < 0.05). [file 12935_2021_2159_MOESM2_ESM.pdf]
